# How to integrate patient and carer perspectives, methodological rigor, and ethics into biomedical research funding

**DOI:** 10.1371/journal.pbio.3003551

**Published:** 2025-12-04

**Authors:** Hella Lichtenberg, Christina Müller, Henk Lindeman, Leila Ali, Anja Minheere, Monique van den Eijnden, Ulrich Dirnagl

**Affiliations:** 1 DLR Projektträger, Division Health, Bonn, Germany; 2 Cognitive Fatigue.eu, IJsselstein, The Netherlands; 3 Faculty of Medicine, McGill University, Montreal, Quebec, Canada; 4 University of Camerino, Macerata, Italy; 5 Open Universiteit, Heerlen, The Netherlands; 6 MIND Platform, Amersfoort, The Netherlands; 7 QUEST Center for Responsible Research, Berlin Institute of Health at Charité Universitaetsmedizin, Berlin, Germany

## Abstract

Patient and carer perspectives, methodological rigor and ethical considerations can all be successfully integrated into the biomedical funding process. This Community Page draws on experiences with ERA-NET NEURON to present a structured, scalable and transferable model for funders to follow.

## Teaser

Patient and carer perspectives, methodological rigor, and ethical considerations can all be successfully integrated into the biomedical funding process. Drawing on experiences with ERA-NET NEURON, we present a structured, scalable, and transferable model for funders to follow.

Robust, trustworthy, and human-centered health research is a cornerstone of modern society and is essential for creating impact, especially amid aging populations, rising rates of chronic health conditions, and declining financial and societal support. By providing the resources necessary for research and through their research policies, funders are pivotal in improving the quality, transparency, and societal value of science. They can foster rigorous methodologies, support data sharing and incentivize research with meaningful scientific and societal impact by setting criteria for proposal selection, evaluation and reporting, and their strategic decisions help shape researcher behavior, institutional practices, and the broader research culture, making them powerful drivers of systemic change toward more trustworthy and impactful science

However, initially promising research findings often do not lead to improvements in healthcare. A substantial proportion of key findings in the biomedical literature are not reproducible, likely owing to flaws in study design, methodology, analysis and reporting. Additionally, research is often not grounded in questions relevant to users such as scientists, patients, and caregivers. Many stakeholders, including academic institutions and funding bodies, acknowledge that a considerable share of investment in biomedical research is ultimately wasted [1].

Against this backdrop, we have been involved in coordinating an effort to enhance the research quality and relevance of the programs of ERA-NET NEURON [2], a transnational network of over 30 funding organizations from Europe and beyond, supporting multidisciplinary and translational research on neurological, psychiatric, and sensory disorders through joint calls for proposals. By aligning national funding strategies and promoting transnational collaboration, NEURON advances the understanding, diagnosis, and treatment of brain-related diseases while strengthening the societal impact of neuroscience. NEURON explicitly aims to fund both preclinical and clinical research, often supporting translational projects up to proof‑of‑concept clinical studies. Its Joint Transnational Calls target the entire value chain from laboratory models to early-stage patient trials.

The decision body of the funding consortium decided in 2017 to initiate and implement changes in the processes towards the improvement of biomedical studies. Consequently, over the past eight years, NEURON broadened its evaluation criteria for proposals to newly include the quality of experimental design and data management, relevance to and feasibility for patients, patient involvement in the projects, and ethical considerations. Patient and carer representatives, along with experts in statistics, ethics, and methodology, became regular participants in the peer review process. Inspired by Canada’s Strategy for Patient-Oriented Research, NEURON involved patients at an early stage in setting research agendas and co-developing funding calls.

Overall, around 30 patient and carer representatives, and European Umbrella Organizations (European Federation of Neurological Associations, EFNA; Global Alliance of Mental Illness Advocacy Networks, GAMIAN) from at least 14 countries contributed as reviewers and/or in the co-creation of the procedures, and 14 of the patient and carer representatives have been continuously active over the years. Patient reviewers were strategically recruited from European patient-led organizations representing diverse neurological or psychiatric conditions, age groups, genders, and countries. Trained by EFNA and other organizations, their number and involvement were based on diversity, equity, and inclusion aspects, lived experience relevant to the research topics, peer review needs and their active engagement in organizations to represent broader patient and carer perspectives.

Initial changes were quickly institutionalized, starting with stricter design requirements in the calls. These led to marked improvements in proposal maturity. NEURON also created a patient engagement (PE) working group with international partners to guide engagement strategies. From 2018 onward, patient and carer perspectives were embedded in specific calls, including participation by advocacy groups and patient observers in peer review sessions.

NEURON expanded its quality initiatives through its role in the European Brain Research Area, supporting open science, reproducibility, and standard-setting. Workshops (both virtual during COVID-19 and in person afterward) trained researchers in key topics including experimental design, neuroethics, data management and patient involvement. These efforts align with a growing international movement to make research more transparent, robust and responsive to societal needs [3–5], and provide a best practice example for engaged funding organizations. By 2022, NEURON had hosted five major workshops involving nearly 300 researchers, including personalized consultations to strengthen study design and reproducibility.

Patient involvement deepened through collaborations with the EFNA (a non-governmental organization that brings together 21 pan-European neurology patient and carer groups), the development of training curricula for patient (and carer) reviewers, and the integration of lay summaries, lectures, and videos to increase public understanding. From 2022, all proposals (clinical and preclinical) were reviewed by trained patient and carer teams using custom evaluation forms. Importantly, patient reviewers often draw on input from patient or family/carer organizations, providing deep knowledge of population-specific needs and health priorities, including the perspective of broader patient populations and encompassing national-level barriers. This means the review is not restricted to the individual reviewer’s lived experience.

In 2025, patient and carer reviewers actively led a symposium workshop for funded projects, reflecting their central role and fostering direct interaction between researchers and patients. These advancements will continue beyond NEURON’s end in 2026 through the European Partnership for BrainHealth, which aims to expand inclusive, quality-driven brain research.

The success of the implementation of the framework for patient involvement, as well as methodological rigor and ethics aspects in NEURON has been constantly monitored by dedicated “lessons learnt” sessions involving funders, patient, and scientific reviewers. Positive feedback, but also modifications—if recommended—were embedded in the processes. Evidence for the general success is provided by the continuation of this framework in the successor initiative, the European Partnership for BrainHealth, which will work with a greater number of funding organizations, thus guaranteeing the sustainability and wider-ranging visibility of the approaches. Furthermore, NEURON has measured success and impact according to key performance indicators (KPIs), for which, e.g., biomedical studies involving patients, are counted selectively. However, since those KPIs are only available after the completion of the projects, distinct results regarding the impact of the framework are still pending.

As a result of our efforts, we have identified several key recommendations (Box 1) that highlight the importance of a step-by-step, collaborative and structured approach to embedding patient and carer perspectives [6–8] and research quality into research funding programs. Key points include co-designing procedures with all relevant stakeholders—such as patient advocates, domain experts (e.g., in biostatistics, ethics, AI), and reviewers—to shape program policies and project evaluation, and using a staged approach to ensure thoughtful integration of diverse perspectives. Continuous feedback from all participants should be used to inform ongoing improvements; a holistic approach should embed PE and quality considerations throughout the entire program lifecycle, from call design to project support and result dissemination. Training is essential for both researchers and patients/carers, particularly in preclinical contexts, to build mutual understanding and improve research design and quality assurance. Capacity building through train-the-trainer models and collaboration with advocacy and academic organizations is encouraged. Finally, clear procedures, agreed documentation and dedicated budgets are necessary to operationalize these commitments effectively.

**Box 1. Key recommendations for embedding patient and carer perspectives in research**

**1. Co-design processes**

Develop all application and evaluation materials, criteria, and training in close collaboration and exchange with stakeholders (e.g., patient and carer representatives).

**2. Empower patient reviewers with tailored application and evaluation processes**

Co-create a lay information template for applicants that prompts researchers to explain their projects in accessible language [7] (see https://doi.org/10.5281/zenodo.16810770). This should cover the research question, methods, expected outcomes, and relevance for patients or carers, including anticipated benefits, risks, timelines, and participant burden. Applicants should also outline the role of patients/carers in designing the study and describe how findings will be communicated to participants and the broader public. In addition, introduce a structured evaluation form (see https://doi.org/10.5281/zenodo.16810770) for patient and carer reviewers complementary to the scientific review. This form guides reviewers to assess the clarity of the lay description, relevance to patient and carer needs, participant burden, feasibility, and the depth of patient/carer engagement. It also covers support provided to participants, data privacy, and dissemination plans. Reviewers provide an overall assessment and actionable suggestions from a patient-centered perspective.

**3. Offer training for researchers and patient and carer reviewers**

Provide structured training to build capacity and shared understanding. For researchers, offer an Open Science Workshop (see https://doi.org/10.5281/zenodo.16810770), ideally spanning more than one day, including sessions on experimental design (e.g., validity, reliability), data management planning, preregistration, ethics, AI, and PE. Include interactive small-group consultations and project-specific feedback. For patient/carer reviewers, implement an interactive training program (see https://doi.org/10.5281/zenodo.16810770) beginning with a pre-training video. Cover research funding basics, the role of PE, evaluation criteria and communication skills. Include breakout discussions, practice evaluations and a mock review panel to simulate real-world decision-making.

**4. Strengthen the interface between ethics and methodology**

Experimental and statistical design, data management plans, pre-registration strategies, open science, and data sharing approaches are key elements of ethical study design (see https://doi.org/10.5281/zenodo.16810770). To ensure rigorous evaluation, the review process must include experts in methodology—especially in study design, statistics, ethics, and AI—alongside patient and carer representatives. Their expertise is crucial for identifying superficial or inadequate responses and ensuring that projects meet high standards of scientific and societal value.

**5. Build sustainable capacity and institutionalize PE**

**Fig 1:**
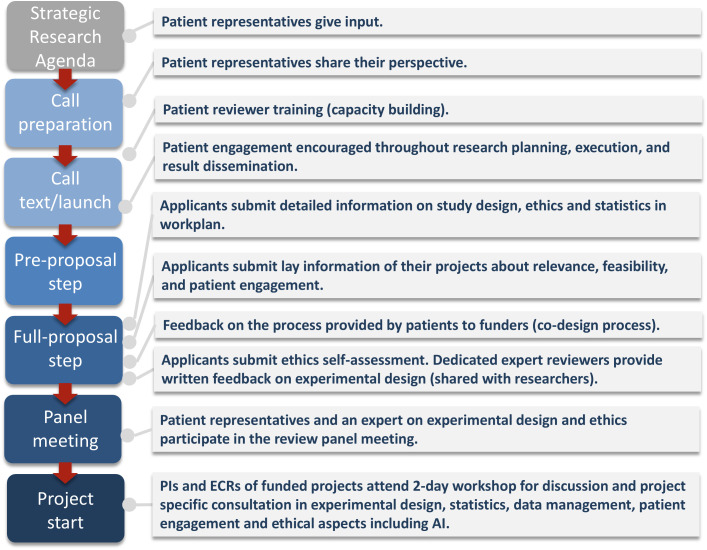
The ERA-NET NEURON program design and evaluation process. Patient involvement and quality assurance elements are integrated into the ERA-NET NEURON program design and evaluation process.

Invest in long-term capacity building through train-the-trainer models and partnerships with patient and family/carer advocacy groups, domain experts in open science, statistics, study design and ethics, and academic societies. PE and research quality should be embedded, not only in application and selection processes, but throughout the entire funding cycle, including project support and dissemination and overarching strategies (Fig 1).

By taking a stepwise and co-creational approach, the perspectives and needs of patient and carer representatives can be meaningfully integrated, even into preclinical research [9]. For a funder, prioritizing both internal validity (e.g., experimental design, data management, statistics) and external validity (i.e., generalizability), together with ethical considerations, is feasible and widely acceptable. This is especially true when these priorities are built into program design and application selection, supported by domain expertise in experimental design and ethics, and reinforced through the education and training of applicants and reviewers.

## Abbreviations

KPIs: key performance indicators
PE: patient engagement
